# Give heart cells a beat: An interactive museum exhibit that synchronizes stem cell–derived cardiomyocytes to visitors’ heartbeat

**DOI:** 10.1016/j.stemcr.2024.01.004

**Published:** 2024-02-08

**Authors:** Juan A. Perez-Bermejo, Samuel J. Reisman, Joyce Ma, Dana Carrison-Stone, Chris Cerrito, Alexandre J.S. Ribeiro, Bruce R. Conklin, Kristina Yu

**Affiliations:** 1 Gladstone Institutes, San Francisco, CA, USA; 2 Exploratorium, San Francisco, CA, USA; 3 Innovative Genomics Institute, Berkeley, CA 94704, USA; 4 University of California, San Francisco Departments of Medicine and Ophthalmology, San Francisco, CA 94143, USA

**Keywords:** iPSC, cardiomyocyte, heart beat, interactive museum exhibit

## Abstract

Science museums play an important role in science education, engaging the public with science concepts and building support for scientific research. Here, we describe Give Heart Cells a Beat, an interactive exhibit that lets museum visitors synchronize the beating of live stem cell–derived cardiomyocytes to their own heart rate in real time. The beat rate of cells accurately matched the beat rate of visitors and responded dynamically to changes such as exercise. Visitor evaluation revealed that engagement with the specimen prompted curiosity in heart biology and stem cells. Give Heart Cells a Beat is the product of a close collaboration between a museum and an academic research laboratory, and to our knowledge, it is the first interactive exhibit to use live human heart cells. We hope this exhibit serves as an example for the implementation of stem cell technology in informal science education and inspires future relationships between academia and public science venues.

## Highlights

• Interactive museum exhibit using live stem cell-derived cardiomyocytes
• Visitors can synchronize the cells’ beat rate to their own heartbeat in real time
• Exhibit prompted visitor curiosity for stem cells and heart biology
• Product of a close collaboration between an academic lab and a science museum

## Teaser

Perez-Bermejo et al. report on an immersive museum exhibit that allows visitors to dynamically synchronize the beating of stem cell–derived cardiomyocytes to their own heartbeat. This is a prime example of how collaboration between an academic lab and a science education center can produce novel ways to communicate current scientific advances to the public.

## Introduction

Recent advances in cell biology, such as the development of induced stem cell technologies, have led to breakthroughs in basic and translational research (Shi et al., 2017) and are increasingly relevant to public life (Aiyegbusi et al., 2020; Critchley et al., 2013; Dasgupta et al., 2014; Longstaff et al., 2013; McNeish et al., 2015; Sayed et al., 2016). This scientific and societal prominence has created an opportunity for scientists and educators to bring these technologies to the public in an engaging and relatable manner and create new types of educational experiences. Museums and science centers are key components of the science education landscape (Bell et al., 2009; 2016; Lander and Gates 2010; Schiele 2009) and provide rich opportunities for reaching a wide and diverse audience (Bell et al., 2009). In addition, there is a growing appreciation for interactive museum exhibits (Allen 2004; Bell et al., 2009; Falk et al., 2004; Pallud 2017) containing real and living samples, rather than recordings or simulations (Allen 2004), to promote visitors’ interest in, engagement in, and understanding of the content. However, the development of interactive biology exhibits featuring live human cells has been significantly limited by cost and availability of reagents, capabilities needed for museums to maintain and display samples such as cell cultures, and lack of mechanisms that enable visitor interaction with microscopic specimens. As a result of the complexity associated with exhibiting cells in culture, cell biology exhibits at science museums have traditionally been limited to noninteractive models, fixed samples, or simulations. Academic research laboratories have been called on to expand their involvement in science education (Miller, 2010) and represent a promising partner in overcoming these challenges (Alpert, 2013).

Here, we report the development and assessment of Give Heart Cells a Beat (GHCB), an exhibit that allows visitors to interact with living human induced pluripotent stem cell (iPSC)–derived cardiomyocytes (iPSC-CMs) by synchronizing the beating of the cells to the visitors’ heart rate. We also report on a visitor evaluation study performed to gauge visitor interest and understanding of the GHCB exhibit and whether it prompted visitors to further consider important scientific and health-related topics. Created via a close and continuing partnership between an academic laboratory and a science museum, GHCB uses human stem cell–derived tissue and demonstrates that stem cell technology can be used to create innovative educational experiences engaging to museum visitors. The exhibit debuted in 2019 and is permanently installed in the biology gallery at the Exploratorium, an interactive museum of science, art, and human perception in San Francisco, California, that welcomes hundreds of thousands of visitors annually. GHCB is placed adjacent to the Exploratorium’s microscope facility in an exhibition area focused on cell biology, alongside other exhibits that contain live samples in close proximity.

## Results

**Figure 1:**
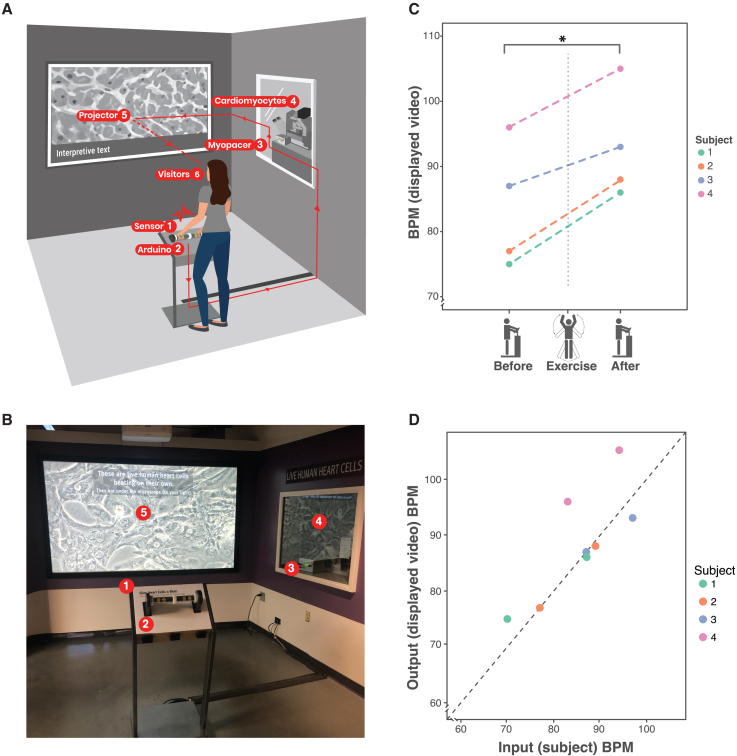
The GHCB exhibit enables visitors to accurately synchronize the beat rate of iPSC-derived CMs to their heart rate in real time (A) Schematic diagram of the functioning of the exhibit. The different parts of the exhibit (1–5) form a circuit that is closed by the visitor (6). (B) Photography of actual exhibit layout. (C) The GHCB exhibit responds to changes in the heart rate of visitors. Displayed beat rate (BPM; obtained by video analysis) of iPSC-CMs in culture synchronized to 4 different users before and after performing light exercise (N = 4, paired t test p = 0.004). (D) Comparison of actual heart rate of the user (measured by a medical-grade heart rate monitor) to the displayed beat rate for the cells in culture (measured by automated analysis of exhibit output video). Poorer correlation for user 4 was probably due to an improper adjustment of the heart rate monitor (linear regression, N = 8, *R*^*2*^ = 0.97, p = 0.0002).

We designed GHCB to allow museum visitors to interact dynamically with cells in culture (Figures 1A and 1B; Videos S1 and S2; Figures S1 and S2). A key design goal was the ability for visitors to interact with live *in vitro* samples in real time. We posited that beating human heart muscle cells, projected in front of the visitor at “human scale,” would be a relatable and compelling “hook” to capture visitor interest. We chose to project living, normally microscopic samples on a wall (Figure 1) and position the visitor in the center of the experience as a way of emphasizing connectedness with the sample (Lam et al., 2019). Although the field of stem cell biology is rapidly evolving and, in some cases, controversial (Aiyegbusi et al., 2020; Critchley et al., 2013; Dasgupta et al., 2014; Longstaff et al., 2013; McNeish et al., 2015; Sayed et al., 2016), the exhibit was designed to focus visitor attention on what is immediately visible and relatable to oneself (stimulating live cells *in vitro* to mirror one’s heartbeat) and possibly provide a bridge to other more challenging concepts such as stem cell biology or cardiology (Durant, 2004; Hine and Medvecky, 2015). In GHCB, an exterior handlebar-style heart rate sensor (Figure S3A) is connected to a pacing electrode that is inserted in a cell culture plate containing iPSC-CMs (Figure S3B) within a microscope chamber. When visitors place their hands on the handlebar, their heart rate is measured and communicated to the submerged electrode, which synchronizes the beating of the cells in culture to the measured stimulus in real time. A live feed from the microscope is projected on a large screen for the visitors to observe. The microscope and cells in an environmental control chamber are inside the museum’s laboratory facility. They are visible through a large glass window to help visitors appreciate the scale and authenticity of the specimen (Lam et al., 2019) (Figure S3C). The interaction of the visitor with the exhibit is guided using an interpretive text overlay (Figure S3D; Table S1) that encourages approach and interaction (e.g., “*These are live human heart cells beating on their own. They are under the microscope (to your right)*.”; “*( …) grasp the handlebar*”), explains the functioning of the exhibit (“*The handlebar senses your heart rate and sends it to the live heart cells under the microscope*.”*)*, and encourages reflection and dynamic interaction (e.g., “*These human heart cells were grown from stem cells in a lab*.”; “*How do these heart cells respond after you do some exercise*?”). An additional wall graphic placed next to the microscope window also explains the exhibit's functioning and the nature of the specimen (Figure S3E).

Video S1. Description of the GHCB exhibit and demonstration of use, related to Figure 1

Video S2. Example of projected cells beating, related to Figure 1

To test the functioning principle of GHCB, we asked a set of volunteers to interact with the exhibit before and after performing a short exercise routine. In all of the cases, we were able to observe a significant increase in the displayed beat rate after exercise (Figure 1C). In addition, we observed a near-perfect correlation between the actual heart rate of users and the beat rate of the cells in the projected video (Figure 1D). This allowed us to conclude that GHCB accurately and sensitively synchronizes the beat rate of iPSC-CMs to the heart rate of the user in real time.

**Figure 2:**
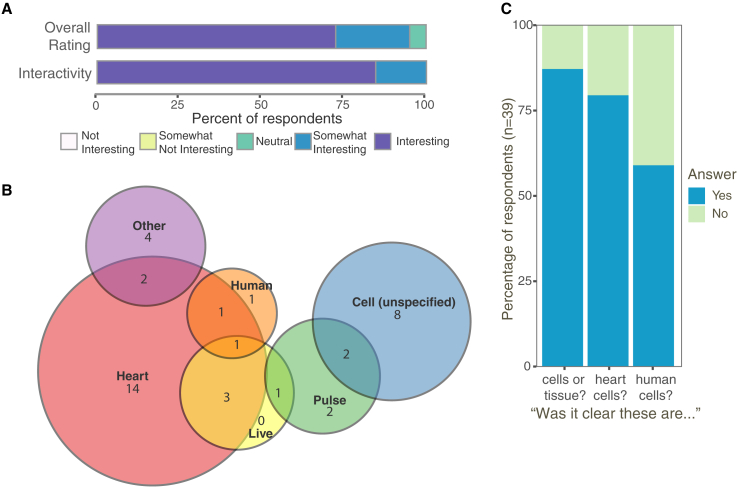
Visitor evaluation (A) Tally of interest and interactivity ratings reported by visitors interviewed after using the exhibit (N = 40 randomly chosen individuals for overall rating, n = 39 for interactivity rating). (B) Venn diagram of self-reported key terms visitors used to describe what they remembered seeing on the screen (N = 39 randomly chosen visitors). (C) Visitor responses when asked directly about what was shown on the screen.

To assess visitors’ reactions to GHCB, we observed unprompted museum visitors as they interacted with the exhibit, then approached and interviewed a subset of those visitors (for detailed results and discussion, see supplementary information 2). GHCB’s “holding time,” a standard metric used to measure engagement (Bell et al., 2009; Griffiths and King, 2008; Lander and Gates, 2010; McLean, 1993), was relatively long compared to the holding time of other exhibits in the museum (median time ∼1 min compared to an average 42 s over 37 exhibits; supplementary information 2), which suggests that visitors found the exhibit engaging. In addition, most of the visitors interviewed reported finding the exhibit interesting (Figure 2A), the two most frequently given reasons being interactivity and the opportunity to see live heart cells (e.g., “[I usually] don’t see heart cells because they are in you”; “[The exhibit] makes it feel very intimate and tangible”). The interviews also revealed that most visitors understood they were looking at heart cells or tissue, with a smaller majority reporting understanding that these cells were of human origin (Figures 2B and 2C). In addition, 90% of visitors reported thinking more about their own hearts (e.g., “I wondered about what condition my heart really is in, and it made me interested in taking more care of my heart.”), whereas 30% of visitors mentioned the technology behind the exhibit (e.g., “How did they do that? How much electricity can you use?”) and 20% talked about stem cells (e.g., “I was just interested in the fact that they were able to recreate human heart cells with stem cells”). Taken together, these findings suggest that GHCB provided an engaging, relatable experience and sparked thoughts about the heart and, to a lesser extent, current biological research concepts such as stem cells and electrophysiology.

**Figure 3:**
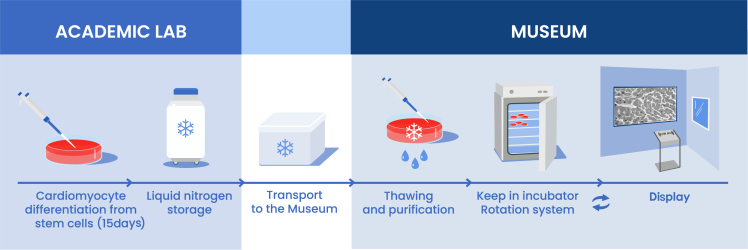
GHCB is an interactive, permanent exhibit enabled by direct collaboration between an academic lab and a museum Stem cell–derived CMs are produced in an academic lab and cryopreserved. When requested, frozen vials are handed over to the museum, where they are thawed and lactate purified (if desired, purification can instead be completed in advance at the academic lab). Cells are kept in an incubator in the museum and rotated in and out of the exhibit to prevent excessive deterioration.

Ultimately, GHCB is made possible by a close collaboration between a state-of-the-art academic stem cell research laboratory, which provides the cell specimen and scientific guidance, and a science museum that provides pedagogical and design expertise and access to a wide public audience (Figure 3). This collaboration is facilitated by the close physical proximity and constant feedback loop between the two institutions. The iPSC-CM cells are produced, differentiated, and stored in the academic lab as part of a routine protocol. Then, frozen vials are transferred to the museum laboratory to be thawed and metabolically purified for use (Tohyama et al., 2013). The postmitotic nature of CMs allows for extended culturing, minimizing labor-intensive replating steps, and the use of consumable materials. In our experience, cells tolerate 4–5 months of intermittent pacing in the exhibit before becoming refractory to pacing, which correlates with the onset of sarcomeric abnormalities (Figure S4). However, up until becoming refractory to pacing, older cells typically displayed clearer synchronization to visitor heartbeat, due to the decrease in spontaneous beat rate as time in culture increased. Typically, the cells allocated for the exhibit were excess cells that did not have a direct experimental use within the academic lab. However, if cells were differentiated explicitly for the exhibit, a single differentiation batch could maintain the exhibit for several years (e.g., four 10-cm dishes yielding ∼40 million lactate-purified iPSC-CMs will allow the exhibit to run for a conservative estimate of 5 years, with an approximate production cost of $500 in reagents and 10 h of labor). Thus, cells can easily be made available to the museum, circumventing otherwise prohibitive costs of purchase. In the case described in this report, the museum leveraged an existing laboratory (with a basic cell culture facility), a microscopy facility, and museum staff with training in cell culture to support the exhibit. In this collaboration, the academic lab benefits from the increased understanding and appreciation of its research by a broad audience, and the museum expands its capabilities and bolsters its mission to communicate cutting-edge scientific and research concepts to the general public.

## Discussion

The GHCB exhibit is a novel educational implementation of stem cell technology and uses the uniquely dynamic behavior (beating) of CMs to enable a real-time interactive experience. The exhibit is a proof-of-concept of the potential for stem cell technology to affect the field of science education (by enabling visual inspection of and interaction with a complex yet relatable cell phenotype), and a pioneering example of the application of biotechnological advances to provide improved tools for science communication. Other exhibits have leveraged live human tissues and cell lines and, occasionally, stem cell–derived cells for public engagement. A notable example is the Tissue Culture & Art Project, which has produced multiple examples of the use of tissue engineering as a form of artistic expression (Catts and Zurr, 2012; Zurr and Catts, 2017). More similar to the GHCB exhibit was the Ergo Sum installation, which used tissue derived from the artist’s own iPSCs (Jarvis, 2018). However, to our knowledge, these have not occurred in an interactive science education center or museum. GHCB provides a layer of interactivity with living cells, positioning the visitor as a unique and critical participant in the experience, which we have observed contributes to visitor engagement and introspection. In addition, because CMs are long-lived and can be imaged and electrically stimulated in a sterile microscope chamber, away from contaminants, GHCB can remain as a permanent exhibit in the museum. The GHCB exhibit was conceived of by Exploratorium staff and Gladstone Institutes scientists and builds upon an existing collaboration that featured a live video of mouse stem cell–derived CMs under a microscope as an early display of the potential of stem cell technology. Currently, GHCB enables a continued exchange of reagents and information between the institutions, allowing for constant refinement of the exhibit and the protocols, and provides a foundation for further interactive exhibit development featuring different stem cell–derived cell types or other state-of-the-art biological research tools and advances.

In this study, we have reported the development of GHCB, the first science museum exhibit to allow dynamic, real-time interaction between museum visitors and cultured human stem cell–derived cells. Our evaluation study with visitors highlights the importance of interactive design to foster engagement with biology content. Comments gathered during evaluation indicate that some visitors found the exhibit personally relevant, prompting them to reflect on how their own bodies function. In future work, visitor interest in GHCB may be leveraged to pique interest in companion exhibits highlighting, for example, other iPSC-derived cell types, the applications of stem cell technology, or exhibits that highlight heart function and physiology (Eisner et al., 2017).

GHCB is the product of an effective and sustained collaboration (> 15 years) between an academic lab and a science education center and an example of how long-term collaborations can produce novel implementations of current technology to communicate scientific advances to the public. We hope that this exhibit inspires other informal science centers and research labs to collaborate and explore ways of developing exhibits or programs that enrich both parties while engaging the public. Although developing and maintaining an exhibit containing live human cells requires specialized infrastructure, similar less resource-intensive activities can be implemented—for example, using live feeds or simulations. As trusted public institutions (Griffiths and King, 2008; Leiserowitz et al., 2010), museums and science education centers are uniquely positioned to collaborate with research labs in raising awareness of the importance of biomedical research. Exhibits such as GHCB can contribute to this task by inviting the public to experience and ask questions about a topic that they would not normally encounter. The GHCB is part of the permanent collection at the Exploratorium museum in San Francisco, where thousands of visitors have already interacted with it.

## Experimental procedures

### Resource availability

#### Lead contact

Further information and requests for resources should be directed to and will be fulfilled by the corresponding authors, Kristina Yu (kyu@exploratorium.edu) and Bruce Conklin (bconklin@gladstone.ucsf.edu).

#### Materials availability

This study did not generate new unique reagents.

#### Data and code availability

This study did not generate new code. Requests for additional data will be fulfilled by the corresponding authors.

### Exhibit design

Upon approaching the exhibit, the visitor triggers a proximity sensor, prompting an on-screen graphic to appear with instructions to grab a hand-activated heart rate sensor (Insta-Pulse). The visitor’s heartbeat is detected by the handheld sensor and sent via an Arduino device to a Myopacer Cell Stimulator (IonOptix), which then delivers a pulse (biphasic, 10 V, 10 ms) to a plate of iPSC-CMs via a submerged 2-prong carbon electrode (IonOptix). The CMs contract in response to each detected beat. A camera captures the beating of the cells and routes it through a program that overlays text instructions to a projector for the visitor to view. As the visitor continues to use the exhibit, the on-screen instructions change, prompting different activities. The Arduino also sends the signal to a haptic feedback system (Uxcell), which sends a vibration pulse to the handlebar in response to each beat, helping visitors monitor their own heart rate and verify the synchronization of the cells. The microscope system (Zeiss Axiovert 200M) is equipped with an environmental control chamber enabling cells to be paced on exhibit for up to 3 days.

### Human iPSC culture and differentiation into CMs

Cells from the WTC human iPSC line, derived from a healthy male subject (Judge et al., 2017) were maintained in mTeSR1 (STEMCELL Technologies) media on growth factor–reduced Matrigel (8 μg/mL, BD Biosciences) and passaged every 3–4 days using Accutase (STEMCELL Technologies). ROCK inhibitor Y-27632 (10 μM, Selleckchem) was added to the media for 24 h after each passage. Cells were differentiated into CMs as described previously (Perez-Bermejo et al., 2021). Briefly, iPSC cultures were given 12 μM CHIR99021 (Tocris) in RPMI 1640 (Gibco) with 2% B-27 supplement without insulin (Gibco) approximately 72 h after plating (day 0). Media was changed to RPMI/B27 without insulin 1 day later, and then RPMI/B27 (without insulin) containing 5 μM IWP2 (Tocris). After another 48 h, the media was changed to RPMI/B27 containing insulin. Fresh RPMI/B27 was exchanged every 3–4 days thereafter. Differentiation success and efficiency was determined visually by the presence of beating cell sheets. On day 15, cells were harvested using 0.25% Trypsin (Gibco) and either replated for lactate purification (see below) or directly frozen in CryoStor media (BioLife Solutions) for later lactate enrichment and plating. Cells were stored in liquid nitrogen tanks until being transferred to museum facilities for thawing.

### Museum & academic laboratory collaboration and training

The implementation of iPSC-derived CMs depended on a close collaboration between the museum and academic lab listed. Conversations between the institutions enabled the exhibit to be designed to leverage the expertise of the academic lab while accommodating the capabilities of the cell culture facility of the museum. Initially, museum staff with relevant scientific backgrounds were trained in CM culture by members of the academic lab, and frequent meetings were arranged to check in on cell status and exhibit performance. Subsequently, museum staff trained one another, consulting with academic lab members when needed. These consultations were facilitated by regular (every 3–6 months) visits to the academic lab to pick up deep-frozen vials to thaw for the exhibit (Figure 3), which allowed for the exchange of feedback, updates on cell behavior, and informal learning and skill exchange.

### Cell thawing, purification, and maintenance on exhibit

Frozen vials of differentiated cells were transferred to the museum laboratory on dry ice. Cells were thawed in 6-well plates. Initially, lactate purification was performed within the academic lab. For subsequent vials, purification was performed at the museum. For lactate purification, CMs were enriched using a previously described metabolic selection method (Tohyama et al., 2013). Briefly, 3 days after plating, media was replaced with DMEM without glucose (Gibco) supplemented with 4 mM lactate (Sigma). Lactate media was exchanged every other day for a total of 6 days. During purification, CMs were monitored for beating, a proxy for culture purity and cell health. Lactate purification was stopped on day 4 if a large decrease in the number of beating cells was observed. Cells were then maintained in RPMI/B27 with 0.5% penicillin-streptomycin (Thermo Fisher) or antibiotic-antimycotic (Thermo Fisher) in Matrigel-coated dishes. If cardiac fibroblasts expanded in culture at a later time, then lactate purification could be repeated to avoid confounding the phenomenon exhibited to visitors.

### Pacing on exhibit

Purified CMs were kept in culture until the autonomous beating rate fell below 60 bpm (this typically took 3 months), although the time depended on the freeze batch. The responsiveness of the cells in the exhibit varied between cell lines and age of the cell plate. When necessary, cells could be manipulated into pacing accurately by lowering the temperature of the chamber from 37°C to 35°C. Additional manipulations could be made by increasing the duration of the bipolar electrical signal from 10 to 20 ms and increasing the voltage of the electrical signal from 10 to 20 V. Exceeding these parameters for long intervals of time led to cell death. When the exhibit was not used by visitors, cells beat autonomously but were not externally paced. Usage rate of the exhibit (and thus pacing rate of the cells) did not appear to affect cell health and viability. Each plate of cells was kept on exhibit for 3 days in media containing l-ascorbic acid antioxidant (212.5 μg/mL, Sigma Aldrich), and then allowed to recover 1–2 weeks without pacing before being used again. By rotating through a group of 6–10 plates in this manner, cell stress was minimized and cultures remained viable for multiple months.

### Beat rate analysis

Randomly selected volunteers were asked to interact with the GHCB exhibit before and after performing exercise (15 side-straddle hops, jumping jacks) while wearing a Kardia (AliveCor) heart rate monitor for actual heart rate tracking. For automated analysis of the beat rate of the video output, video clips recorded from the exhibit screen were analyzed using the Pulse Video Analysis platform (Maddah et al., 2015) (Dana Solutions).

### Visitor evaluation

A total of 62 randomly chosen museum visitors were observed, 40 of whom were interviewed. For a detailed discussion on methodology and results, see supplementary information 2: Visitor Research and Evaluation for Give Heart Cells a Beat Exhibit. For the analysis of key descriptors in the visitor interviews, comments were manually annotated and classified. The term “cells-unspecified” refers to answers that acknowledged seeing cells without any other descriptor, and “other” was used for answers that did not fit in previous categories (“an image,” “blood,” or simply “weird stuff”). Data for Likert scores and key terms were plotted using R (version 3.5.3).

### Immunofluorescent staining of sarcomeres for qualitative evaluation of cell health

Cell plates from the exhibit were fixed in 4% paraformaldehyde for 15 min at room temperature. They were then washed with PBS containing 0.1% Triton X-100 (PBS-T), then blocked in 5% BSA (Sigma-Aldrich) in PBS-T at room temperature for 1 h. For sarcomere imaging, α-actinin antibody (A7732, Sigma-Aldrich) was then diluted in 5% BSA solution and incubated overnight at 4°C. Cells were washed in PBS-T and then incubated with secondary antibody (Alexa Fluor 594 goat anti-mouse immunoglobulin G; Molecular Probes) diluted in 5% BSA solution for 1 h at room temperature. Nuclei were stained using DAPI (Vector Laboratories) and cells were imaged using a BZ-X700 microscope (Keyence).

### Ethical approvals and consent

The WTC iPS cell line used in this study was originally derived in the laboratory of Prof. Bruce Conklin, with supervision and approval by the University of California, San Francisco (UCSF) institutional review board (IRB) protocol 10–02521. The use of this cell line by the Exploratorium is enabled by the UCSF Committee on Human Research, which allows for the transfer of iPSCs and derived tissue. The visitor study conducted as part of this work was done at the Exploratorium according to IRB FWA00028642.

## Supplemental information

Document S1. Figures S1–S4 and Table S1

Document S2. Article plus supplemental information
